# Fabrication and Characterization of Chicken- and Bovine-Derived Chondroitin Sulfate/Sodium Alginate Hybrid Hydrogels

**DOI:** 10.3390/gels8100620

**Published:** 2022-09-28

**Authors:** Yaqi Zhao, Yan Li, Tianchan Lan, Baowei Wang, Ming Huang, He Huang, Changming Qiao, Jingxin Sun

**Affiliations:** 1 College of Food Science & Engineering, Qingdao Agricultural University, Qingdao 266109, China; 2 National R&D Branch Center for Poultry Meat Processing Technology, Nanjing Huangjiaoshou Food Sci. & Tech. Co., Ltd., Nanjing 211226, China; 3 Shandong New Hope Liuhe Co., Ltd., Qingdao 266000, China; 4 Zhucheng Waimao Co., Ltd., Zhucheng 262200, China; 5 Qingdao Special Food Research Institute, Qingdao 266109, China

**Keywords:** chondroitin sulfate, hydrogel, characterization, sodium alginate, hybrid

## Abstract

The physicochemical properties and microstructure of hybrid hydrogels prepared using sodium alginate (SA) and chondroitin sulfate (CS) extracted from two animal sources were investigated. SA-based hybrid hydrogels were prepared by mixing chicken- and bovine-derived CS (CCS and BCS, respectively) with SA at 1/3 and 2/3 (*w*/*w*) ratios. The results indicated that the evaporation water loss rate of the hybrid hydrogels increased significantly upon the addition of CS, whereas CCS/SA (2/3) easily absorbed moisture from the environment. The thermal stability of the BCS/SA (1/3) hybrid hydrogel was higher than that of CCS/SA (1/3) hybrid hydrogel, whereas the hardness and adhesiveness of the CCS/SA (1/3) hybrid hydrogel were lower and higher, respectively, than those of the BCS/SA (1/3) hybrid hydrogel. Low-field nuclear magnetic resonance experiments demonstrated that the immobilized water content of the CCS/SA (1/3) hybrid hydrogel was higher than that of the BCS/SA (1/3) hybrid hydrogel. FTIR showed that S=O characteristic absorption peak intensity of BCS/SA (2/3) was obviously higher, suggesting that BCS possessed more sulfuric acid groups than CCS. SEM showed that the hybrid hydrogels containing CCS have more compact porous microstructure and better interfacial compatibility compared to BCS.

## 1. Introduction

Hydrogels are hydrophilic three-dimensional networks formed via chemical or physical crosslinking between synthetic or natural polymers that can be used to deliver drugs in biological environments [1]. Because of their high water content, good biological swelling, and good biocompatibility, hydrogels can be used in clinical and experimental medicine [2]. Furthermore, owing to their excellent properties, hydrogels are widely used in agriculture, the health and pharmaceutical industries, and for tissue engineering, diagnosis, cell fixation, and biomolecular and cell separation [3].

Many studies have demonstrated that because of their porous three-dimensional network structure, hydrogels can be widely used in medicine as carriers for controlled drug delivery [4]. Drugs can be loaded into the porous structure of hydrogels, and small macromolecules can be diffused gradually throughout the gel network [5]. The porous structure of hydrogels promotes the combination of biological active agents with swelling water. This causes the hydrogel network structure to expand, thus promoting release of encapsulated materials [6].

Hydrogels used in biological applications should be non-toxic, biocompatible, and biodegradable. Therefore, hydrogels comprising polysaccharides, such as sodium alginate (SA), chitosan, Arabic gum and chondroitin sulfate (CS), present multiple advantages. owing to their inherent biocompatibility, high water content, and molecular structure similar to that of the natural extracellular matrix [7]. CS, a complex linear anionic heterosaccharide, a sulfated glycosaminoglycan formed by the polymerization of disaccharide units of D-glucuronide and N-acetylamino galactose. As a major component of the extracellular matrix (ECM), CS has anti-oxidant, anti-atherosclerotic, anti-thrombotic and immunogenic properties. Moreover, CS is biodegradable and biocompatible, therefore, it is a good biomaterial with wide applications in tissue engineering. CS can be used to prepare low-toxic, highly biocompatible and reproducible hydrogels and improve their porous network structure [8]. As new biomaterials, CS-based hydrogels have received considerable attention and have been widely used in tissue engineering, drug delivery, cell therapy, and other biological applications [9]. When added to biomaterials, CS can be used to repair bone tissue and promote bone tissue reconstruction. CS-containing bioactive coatings can trigger the growth of vascular cells and inhibit apoptosis [10]. In summary, CS-based biomaterials can be used to replace and regenerate damaged cartilage, bone, skin and nerve tissue [11].

SA, a natural linear polysaccharide formed by β-D-mannuronic acid and α-L-gulonuronic acid linked by 1,4-glycosidic bonds, which can react with divalent cations to prepare gels, can be used in biomedical applications [12]. CS has been used to modify SA-based hydrogels through the coordination of calcium ions between the CS and SA molecules in the absence of chemical cross-linking agents. Physical cross-linking formed by Ca^2+^ coordination has certain advantages over chemical cross-linking, because the use of chemical cross-linking agents can lower the biocompatibility of hydrogels and cause toxicity, and the application of hydrogels containing additional chemical cross-linking agents in the biomedical field can be greatly limited [13]. Hence, CS/SA-based hydrogels without cross-linking agents can be used as drug delivery systems.

CS was isolated from various tissues of vertebrate and invertebrate animals. CS presents a wide range of functional properties because of its heterogeneous structure and its various physical and chemical properties, which depend on the species and tissue it is isolated from. The efficacy of CS depends on its source; therefore, the source of CS is the critical factor determining its biological activity. Currently, most of the CS widely used in the market is extracted from bovine cartilage. Rani et al. have shown that CS extracted from chicken cartilage is a cheaper and sustainable raw material that can be used to develop efficient drug delivery vehicles [14]. However, researchers have not yet determined whether the properties of hybrid hydrogels prepared using CS from various animal sources depend on the species CS is isolated from. In this study, we compared the properties of hybrid hydrogels comprising chicken- and bovine-derived CS (CCS and BCS, respectively) and SA by evaluating their evaporation water loss rates, swelling rates and subjecting them to thermogravimetric analysis (TGA), texture analysis, scanning electron microscopy (SEM), Fourier transform infrared spectroscopy (FTIR) and low-field nuclear magnetic resonance (LF-NMR) experiments.

## 2. Results and Discussion

### 2.1. Characterization of CCS and BCS Particles

#### 2.1.1. Particle Size Distribution

**Figure 1:**
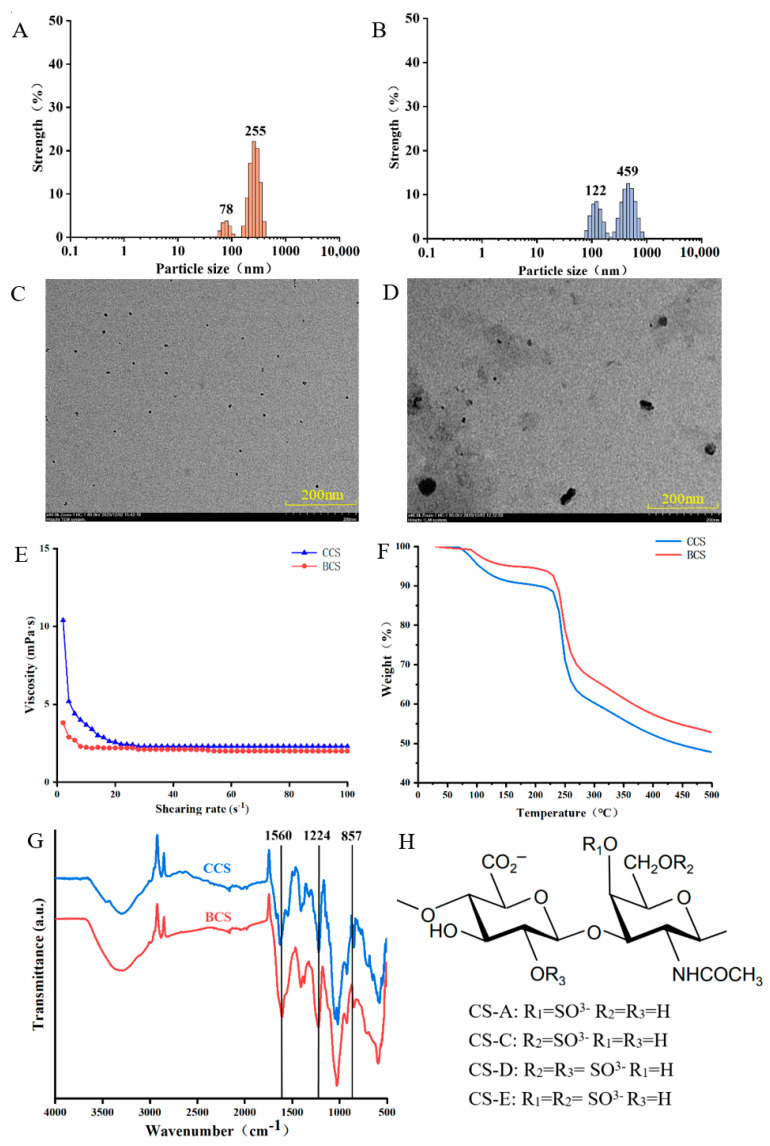
Characteristics of the CCS and BCS particles. Particle size distribution of (**A**) CCS and (**B**) BCS. The average particle size for each peak is marked. TEM images of (**C**) CCS and (**D**) BCS. (**E**) Viscosity curves of CCS and BCS in the shear rate range of 0–100 s^−1^. (**F**) TGA curves of CCS and BCS. (**G**) FTIR spectra of CCS and BCS. (**H**) Structural formula of CS.

Figure 1A,B shows the difference in particle size between CCS and BCS based on dynamic light scattering (DLS). The diameters of the CCS particles were 255 ± 18.2 nm (22.1%) and 78 ± 3.4 nm (3.8%), whereas the diameters of the BCS particles were 459 ± 10.68 nm (12.5%) and 122 ± 6.32 nm (8.48%). CS is a complex heterogeneous polysaccharide; therefore its various charge density and particle size affect its chemical properties, pharmacological activity, and biocompatibility. CS samples with small particle size promote cell adhesion and growth. The particle size distribution of CS isolated from chicken keel presented two peaks at 255 and 44 nm, and the peak at 44 nm was attributed to the residual peptides formed during CS production [14]. In this study, the uneven particle size distribution of CS was attributed to the residual peptides formed during enzymatic hydrolysis. Previous studies demonstrated that CCS particles were smaller than BCS particles; therefore, CCS is more suitable for applications in the food and pharmaceutical industries and other fields [15].

#### 2.1.2. Transmission Electron Microscope (TEM) and Rheological Properties

Figure 1C,D show the TEM images of CCS and BCS particles, respectively. The CCS particles were small and uniformly distributed, whereas the BCS particles were large and uneven. CS samples isolated from different animal sources using different extraction methods present different particle sizes. Studies have demonstrated that CS particles are spherical. This was ascribed to the highly branched structure of CS. Moreover, CCS presented a relatively uniform branched chain distribution, whereas BCS presented a complex branched structure.

Figure 1E shows the relationship between the shear rate and viscosity of aqueous solutions of CCS and BCS. The initial viscosities of the CCS and BCS aqueous solutions were 10.4 and 3.8 mPa·s, respectively. Upon increasing shear rate, the viscosities of all the CS aqueous solutions in this study decreased gradually until they became dynamically stable. The balanced viscosities of CCS and BCS were 2.3 and 2.0 mPa·s, respectively. The viscosity of CCS was higher than that of BCS, because water molecules were closely bound to CCS, resulting in the poor fluidity and high viscosity of the CCS solution. Kakkar and Madhan determined that the higher the viscosity of a solution, the more stable the solution is. CS samples with low viscosity are more suitable for injections, eye drops, functional drinks and oral liquid preparations, whereas CS samples with high viscosity are more suitable for thickeners and stabilizers [16].

#### 2.1.3. Thermogravimetric Analysis (TGA)

TGA is widely used to evaluate the thermal behavior and decomposition modes of polymers [16]. Figure 1F shows the TGA profiles of CCS and BCS. The mass loss of CS occurred in two primary stages. The first mass loss stage occurred between 70 and 160 °C, and the mass loss rates of CCS and BCS were 9 and 5%, respectively. The mass loss during the first stage was attributed to the loss of CS water. The second mass loss stage occurred between 230–280 °C, and the mass loss rates of CCS and BCS were 38 and 32%, respectively. The mass loss during the second stage was ascribed to the decomposition of CS. The temperatures at which CCS and BCS reached a mass loss rate of 45% were 360 and 440 °C, respectively. Upon increasing the temperature to 500 °C, the mass loss rates of CCS and BCS were 53 and 48%, respectively. Therefore, the thermal stability of BCS was higher than that of CCS, suggesting that BCS is more suitable for applications that involve heat treatment in the food and drug industries.

#### 2.1.4. Fourier Transform Infrared (FTIR)

The peaks at approximately 3400 cm^−1^ in the FTIR spectrum of CS corresponded to the overlapping stretching vibrations of −OH and N−H, and the peak at approximately 2930 cm^−1^ corresponded to the C−H stretching vibration of the methyl or methylene groups of CS (Figure 1G). The peak at 1636 cm^−1^ corresponded to the amide bands, and the peak at approximately 1560 cm^−1^ represented the −NH band, indicating the presence of −NH−C=O groups in the structure of CS [17]. The peak at 1224 cm^−1^, which corresponded to the stretching vibrations of the S=O bonds of the sulfate groups, is a characteristic absorption peak of CS. According to its structural formula CS can be classified into CS-4 (CS-A), CS-6 (CS-C), CS-2, 6-sulfate (CS-D) and CS-4, 6-sulfate (CS-E) (Figure 1H). Peaks at 857 and 826 cm^−1^ were observed in the FTIR spectra of CCS and BCS, respectively. Studies have indicated that peaks at approximately 857 and 826 cm^−1^ are specific to CS-A, and CS-C, respectively [18]. Johanne et al. demonstrated that CS-A accounted for 61% of BCS, which was similar to the content of CS-A in a standard CS sample [19]. In this study, a peak at 857 cm^−1^ was observed in the FTIR spectra of CCS and BCS, indicating that both types of CS contained CS-A.

### 2.2. Characterization of Hybrid Hydrogels

#### 2.2.1. Evaporation Water Loss Rate

**Figure 2:**
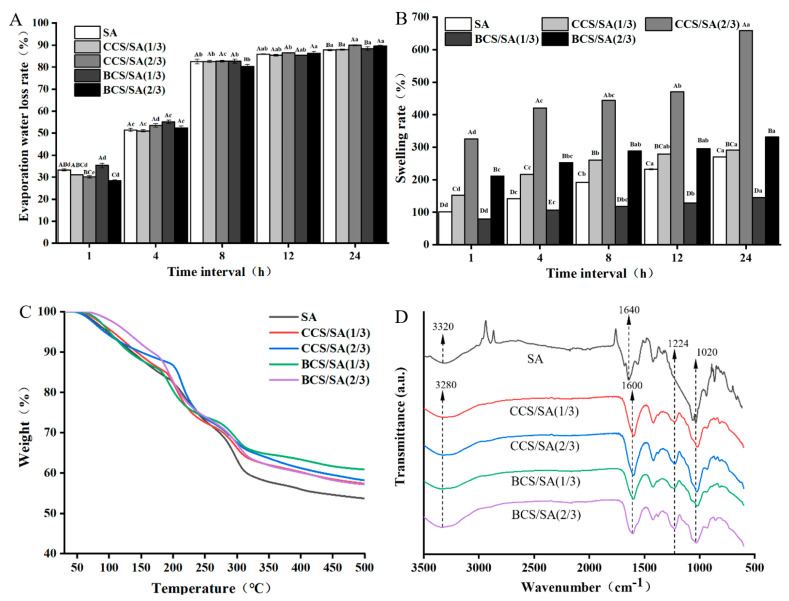
(**A**) Evaporation loss rate profiles, (**B**) swelling rate profiles, (**C**) TGA curves, and (**D**) FTIR spectra of the SA, CCS/SA (1/3), CCS/SA (2/3), BCS/SA (1/3), and BCS/SA (2/3) hydrogels. Different uppercase letters (A–E) indicate significant differences between hydrogels within the same time interval (*p* < 0.05), and different lowercase letters (a–e) indicate significant differences for the same hydrogel over different time intervals (*p* < 0.05).

The evaporation water loss rates of the SA, CCS/SA (1/3), CCS/SA (2/3), BCS/SA (1/3), and BCS/SA (2/3) hydrogels after 1 h at 37 °C were 33.37, 31.00, 30.20, 35.40, and 28.35%, respectively (Figure 2A). After 8 h, the evaporation water loss rates of the hydrogels were high (≥80%). After 24 h, the evaporation water loss rates of the SA, CCS/SA (1/3), CCS/SA (2/3), BCS/SA (1/3), and BCS/SA (2/3) hydrogels reached 87.80, 87.97, 89.92, 88.41, and 89.66%, respectively. The evaporation water loss rates of the hydrogels did not exceed 90% after 24 h. The evaporation water loss rates of CCS/SA (2/3) and BCS/SA (2/3) were significantly higher than those of other hydrogels after 24 h (*p* < 0.05), indicating a CS concentration-dependent effect. The loss of water in the hydrogels at 37 °C was attributed to the evaporation of free water within the hydrogels and formation of pores of different sizes in the hydrogels, which promoted water flow and evaporation [20]. Some studies have demonstrated that the water loss of hydrogels can also be attributed to the charge and polar interactions between polymers and cross-linking density [21].

#### 2.2.2. Swelling Rate

The water content of hydrogels plays a critical role in gel integrity, solubility, and diffusion. Hydrogels absorb water and swell in aqueous media [22]. This is attributed to the adsorption mechanism of hydrogels and diffusion between the polymer network and external solution [23]. The swelling ability of hydrogels is affected by the cross-linking density between polymers, number of hydrophilic groups, and mechanical properties of the polymer networks.

The swelling rate of the BCS/SA (1/3) hydrogel was significantly lower than that of the SA hydrogel within 24 h, and the swelling rates of the other CS-containing hydrogels were higher than that of the SA hydrogel (Figure 2B). The CCS/SA (2/3) hydrogel absorbed the most moisture from the surrounding media within 24 h, and its swelling rate was significantly higher than that of the other hydrogels (*p* < 0.05). CS is a type of macromolecular polysaccharide that contains hydrophilic groups, such as sulfate, carboxyl, and hydroxyl groups. These groups can enhance the interactions between the polymer and water, thus increasing the water absorption capacity and swelling ability of CS/SA hydrogels [24]. Ponsubha and Jaiswal reported that the pore number and pore size of hydrogels increased with CS content, and the water absorption capacities of CS-containing hydrogels were higher than those of CS-free hydrogels [20]. Khalid et al. demonstrated that upon increasing the CS content of hydrogels, the number of ionized groups increased; this promoted the electrostatic repulsion between the ionized groups and increased the swelling rate of the hydrogel [25]. These results indicated that unlike the BCS-based hydrogels, the CCS/SA (2/3) hydrogel presented a better gel matrix, a higher cross-linking density, and more micropores; therefore its ability to absorb moisture from the surrounding medium was higher.

#### 2.2.3. Thermogravimetric Analysis (TGA)

The SA, CCS/SA (1/3), CCS/SA (2/3), BCS/SA (1/3), and BCS/SA (2/3) hydrogels lost mass over three stages (Figure 2C). During the first stage 60–200 °C, the mass loss rates of the SA, CCS/SA (1/3), CCS/SA (2/3), BCS/SA (1/3), and BCS/SA (2/3) hydrogels were 17, 16, 13, 15, and 12%. The mass loss during the first stage was attributed to water evaporation from the polymer chains of the hydrogels During the second stage 195–315 °C, the mass loss rates of the SA, CCS/SA (1/3), CCS/SA (2/3), BCS/SA (1/3), and BCS/SA (2/3) hydrogels increased to 40, 35, 34, 33, and 36%, respectively. The mass loss during the second stage, was attribute to the primary chain of the polysaccharide, breaking down [26]. Upon further increasing the temperature, the third stage of degradation was reached and the hydrogel was completely degraded. During the third stage, the weight loss of the hydrogels was caused by the fracture of the polymer skeletons [27].

At 500 °C, the mass loss rates of the SA, CCS/SA (1/3), CCS/SA (2/3), BCS/SA (1/3), and BCS/SA (2/3) hydrogels were 46, 42, 41, 38 and 42%, respectively. The mass loss rate of the SA, CCS/SA (1/3), CCS/SA (2/3), BCS/SA (1/3), and BCS/SA (2/3) hydrogels was 35% at 299, 315, 351, 392 and 321 °C, respectively. These results demonstrated that the thermal stability of the CS-containing hydrogels was superior to that of the SA hydrogel, and that the addition of CS to SA improved the thermal stability of the hydrogels. The BCS/SA (1/3) hydrogel exhibited the highest thermal stability. Khalid et al. prepared CS and 2-acrylamido-2-methylpropane sulfonic acid hydrogels and confirmed that the thermal stability of the CS-containing hydrogels was higher than those of the hydrogels fabricated using only CS or 2-acrylamido-2-methylpropane sulfonic acid [25], which was consistent with our results.

#### 2.2.4. Fourier Transform Infrared (FTIR)

The FTIR spectra of the SA, CCS/SA (1/3), CCS/SA (2/3), BCS/SA (1/3), and BCS/SA (2/3) hydrogels are shown in Figure 2D. The FTIR spectrum of the SA hydrogel included a wide band at approximately 3320 cm^−1^, which was ascribed to the −OH stretching vibration, bands at 1640 and 1428 cm^−1^, which corresponded to the symmetric and asymmetric stretching vibrations of carbonyl C=O and a band at 1036 cm^−1^, which was attributed to the C−O−C stretching vibration. The stretching vibration bands of N−H and −OH overlapped at approximately 3280 cm^−1^, with a peak at 3310 cm^−1^. The characteristic peak at 1640 cm^−1^ was attributed to the stretching vibration of the amide group [28]. The stretching vibration of carbonyl C=O emerged at approximately 1600 cm^−1^, and the stretching vibrations of the C–O and −OH were observed bands at approximately 1020 and 1430 cm^−1^, respectively. Upon increasing the CS content of the hydrogels, the intensity of the stretching strength of the −OH groups increased [29].

A peak at 1224 cm^−1^, which is attributed to the sulfate groups of CS, was observed in the FTIR spectra of the CS/SA hybrid hydrogels. This demonstrated that CS/SA hybrid hydrogels contained sulfidoyl groups, and CS was present in the hydrogels. Moreover, a cross-linking reaction occurred between CS and SA. The intensities of the characteristic peaks in the FTIR spectra of the CCS/SA (1/3) and BCS/SA (1/3) hybrid hydrogels with low CS contents were comparable; however, the intensities of the absorption peaks in the FTIR spectrum of BCS/SA (2/3) were significantly higher than those in the FTIR spectrum of CCS/SA (2/3). This indicates that crosslinking was stronger in the hydrogels with a higher BCS content. Khalid et al. demonstrated that the peak at 1225 cm^−1^ in the FTIR spectrum of CS corresponded to the stretching vibration of the S=O sulfate group, confirming that this is the characteristic peak of CS [25]. Crispim et al. synthesized polyvinyl alcohol (PVA) and chondroitin sulfate hydrogels. Semi-interpenetrating network hydrogels were formed by cross-linking of PVA, and CS maintained a linear morphology in the matrix [28]. Therefore, the FTIR spectra of the PVA/CS and PVA hydrogels were comparable. However, no distinct peak change were observed between the FTIR spectra of the SA, CCS/SA (1/3), CCS/SA (2/3), BCS/SA (1/3), and BCS/SA (2/3) hydrogels in this study. This demonstrated that the cross-linking reaction between CS and SA was a physical process, and no chemical cross-linking occurred between the polymers [30].

#### 2.2.5. Texture Analysis

Texture analysis can rapidly reproduce the physical properties of the hydrogels surface, and mechanical parameters, such as hardness, adhesiveness, cohesiveness, and resilience, can be evaluated. These parameters reflect the minimum force required for hydrogel recovery, expandability, and ability to restore the original hydrogel structure [29]. The mechanical properties of hydrogels affect their ability to serve as biological tissue scaffolds. It is critical to balance material porosity with mechanical strength when developing tissue engineering scaffolds for cartilage. To support cartilage tissue regeneration at the implantation site, hydrogels should present a porous structure with sufficient mechanical strength [31].

**Figure 3:**
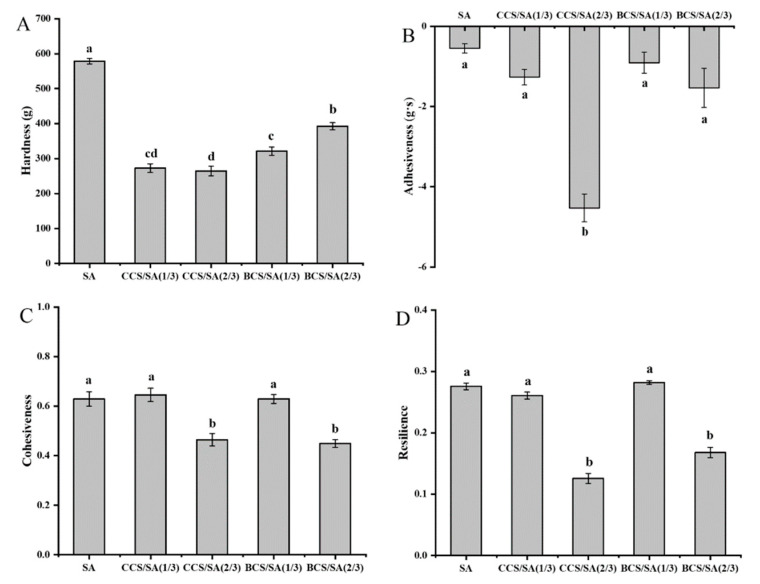
Textural properties of the SA, CCS/SA (1/3), CCS/SA (2/3), BCS/SA (1/3), and BCS/SA (2/3) hydrogels: (**A**) Hardness. (**B**) Adhesiveness. (**C**) Cohesiveness. (**D**) Resilience. Different lowercase letters (a–d) indicate significant differences (*p* < 0.05).

The hardness of the SA hydrogel (578.61 ± 7.94 g) was significantly higher than those of the CS-containing hydrogels (*p* < 0.05) (Figure 3A). The hardness of the CCS/SA (2/3) hydrogel (264.52 ± 11.65 g) was the lowest among the hydrogels in this study. Hydrogel hardness depends on the residence time at the application site. The residence time at the application site decreased with increasing hydrogel hardness. The lower the hydrogel hardness, the weaker the force required to recover from the container, which favors applications of hydrogels as coating materials [32]. The absolute adhesiveness of the CCS/SA (2/3) hydrogel (−4.53 ± 0.35 g·s) was significantly higher than those of other hydrogels in this study (*p* < 0.05). Furthermore, the adhesiveness of the CCS/SA hydrogels increased with increasing CCS concentration. No significant differences (*p* > 0.05) were observed between the adhesiveness of BCS/SA hydrogels. Previous studies have demonstrated that hydrogels with higher adhesiveness present longer retention times at the application site [16]. The cohesiveness and resilience of the CCS/SA (2/3) and BCS/SA (2/3) hydrogels were significantly lower than those of the other hydrogels (*p* < 0.05). Cohesiveness determines the ability of a hydrogel to reconstruct after use. The higher the cohesiveness, the greater the structure restoration ability. Resilience is the ability of a substance to restore to its original position after compression. The hydrogels with higher CS contents presented lower cohesiveness and resilience than those with lower CS contents. This was attributed to the large pores of the CCS/SA and BCS/SA hydrogels. An ideal hydrogel should exhibit easy spreading, considerable elasticity, and high mechanical strength. Based on the hardness, adhesiveness, cohesiveness, and resilience of the hydrogels in this study, it was concluded that the CCS/SA (2/3) hydrogel presented the best texture among the hydrogels in this study.

#### 2.2.6. Microstructure Analysis

**Figure 4:**
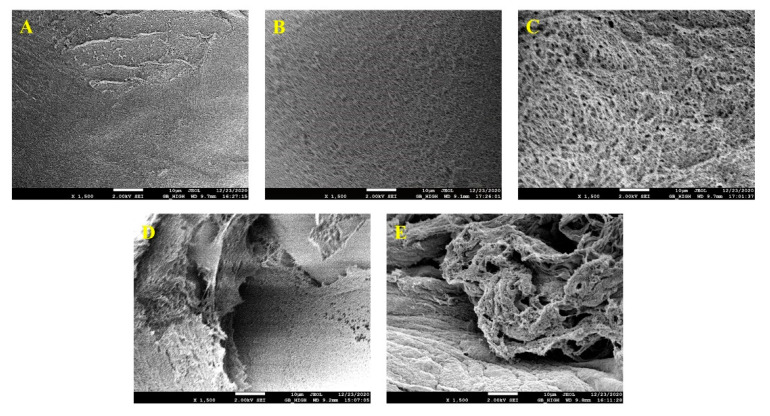
SEM images of (**A**) SA, (**B**) CCS/SA (1/3), (**C**) CCS/SA (2/3), (**D**) BCS/SA (1/3), (**E**), and BCS/SA (2/3) at a magnification of 1500×. The scale bar is 10 µm.

The pores of the SA, CCS/SA (1/3), CCS/SA (2/3), BCS/SA (1/3), and BCS/SA (2/3) hydrogels can be observed in their corresponding SEM images (Figure 4A–E, respectively). The CS-containing hydrogels presented a porous structure, their pores were larger than those of SA, and their three-dimensional network structure was more distinct. The surfaces of the BCS/SA (1/3), and BCS/SA (2/3) hydrogels presented cracks and were rough (Figure 4D,E, respectively). The surfaces of the CCS/SA (1/3), and CCS/SA (2/3) hydrogels were uniform, dense, smooth, and crack-free, and the number and size of the pores on the surface increased with increasing CCS content (Figure 4B,C, respectively). The pores in the structures of hydrogels promote cell migration. Moreover, the larger pores promote cell proliferation and growth, whereas the small pores promote the transfer of nutrients embedded in hydrogels. SEM analysis revealed that the hybrid hydrogels presented highly porous structure with higher gel density and larger pores than those of the SA hydrogel. This structure is anticipated to be conducive to cell growth, reproduction, and migration. The three-dimensional network structure of hydrogels plays a critical role in cell migration. The compatibility of the CCS/SA hydrogels was higher than that of the BCS/SA hydrogels; therefore, the CCS/SA hybrid hydrogels are anticipated to be more conducive to cell growth and transfer than the BCS/SA hybrid hydrogels in practical applications.

An increase of the CS content of hydrogels improved hydrogel porosity and rendered the hydrogels more permeable. Scaffolds used in cartilage tissue engineering should be highly porous and present interconnected pore structures to allow cells to attach to them and multiply [33]. Singh et al. demonstrated that the average pore size of a scaffold fabricated using chitosan and CS was larger than that of a scaffold comprising chitosan alone, and scaffolds with higher porosity are suitable for cell migration, proliferation, gas exchange, and influx, and outflow of nutrients and toxic by-products [34].

#### 2.2.7. Low Field-Nuclear Magnetic Resonance (LF-NMR)

**Figure 5:**
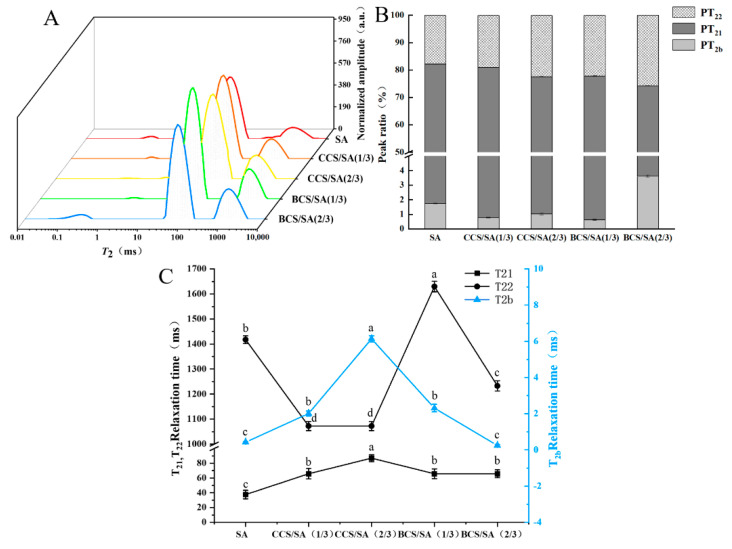
Water distribution in the SA, CCS/SA (1/3), CCS/SA (2/3), BCS/SA (1/3), and BCS/SA (2/3) hydrogels. (**A**) T2 relaxation time distribution. (**B**) Relative composition of water types. (**C**) T2 relaxation time profiles based on the corresponding top peaks. Here, PT2b, PT21, and PT22 are the relative percentages of bound water, immobilized water, and free water, respectively. Different lowercase letters (a–d) indicate significantly different mean values (*p* < 0.05).

Water population and distribution play a critical role in hydrogels, supporting their integrity, solubility, and promoting substance diffusion [3]. LF-NMR can be used to evaluate the flow characteristics of different water types in hydrogel network structures and the interactions between water molecules and macromolecules. Different horizontal relaxation times correspond to different types of movement of water molecules [35]. The shorter the T_2_ relaxation time of water molecules, the lower the mobility of water molecules and the higher the binding degree of the corresponding water molecules [36]. The water present in hydrogels can be divided into bound water, immobilized water and free water. Bound water is strongly bound to the functional groups of the polymer; the binding force between immobile water and the polymer is weak, and therefore no distinct binding occurs between free water and the polymer [37]. The water relaxation distributions of the CCS/SA and BCS/SA hybrid hydrogels are shown in Figure 5A. Three peaks emerged in the range of 1–10,000 ms. According to the relationship between the movement ability of water molecules and T_2_ values, the peaks in the ranges of 0.1–10 ms, 15–500 ms, and >500 ms were classified as bound water (T_2b_), immobilized water (T_21_), and free water (T_22_), respectively.

The T_21_ values of the hybrid hydrogels were significantly higher than that of the SA hydrogel (Figure 5C). The T_21_ value of the CCS/SA (2/3) hydrogel was the largest, which was attributed to the increase in the number of pores of the three-dimensional network structure of the hydrogel resulting in a decrease in the binding strength between water molecules and the polymer chains, wide range of water distribution, improvement in the fluidity of the water immobilized inside the hydrogel, and gradual diffusion of water bound to the polymer outside of the hydrogel. The PT_21_ value of CCS/SA (1/3) was significantly higher than that of other hybrid hydrogels, and the PT_22_ values of the hybrid hydrogels were significantly lower than those of other CS-containing hydrogels (Figure 5B). This indicates that the relative content of immobilized water in the CCS/SA (1/3) hydrogel was higher than that of free water.

## 3. Conclusions

In this work, whether hydrogels prepared from CS of chicken cartilage and bovine cartilage would exhibit different characteristics due to species differences was explored. The physicochemical and structural characteristics of CCS/SA and BCS/SA hybrid hydrogels were investigated. The results showed that the addition of CCS and BCS to SA increased the pore size of the hydrogel and promoted the flow of water, leading to an increase in the evaporative water loss rate and swelling rate of the hydrogel. CCS/SA (2/3) had the highest swelling rate and formed large and dense hydrogel pores. The texture of hydrogels affects their application as biomaterials, and the results showed that CCS/SA (1/3) showed better spreadability, optimal stiffness and structural recovery, and was more suitable for use as a scaffold for biomaterials and cartilage tissue engineering. Thermogravimetric analysis showed that the increase of chondroitin sulfate increased the thermal stability of the hydrogels, with BCS/SA (1/3) having the highest thermal stability. The results of IR spectral analysis showed that all CS/SA-based hydrogels showed the characteristic peaks of S=O, indicating that CS could be successfully incorporated into the hydrogels. It is shown that CS does not participate in any chemical interaction in the mixed system of SA-Ca^2+^, so no new peaks appear in CS/SA-based hydrogels [7]. As observed by scanning electron microscopy, the increase of CS ratio causes the hydrogel to have more pores, and the surface of CCS/SA hydrogel is dense and smooth without cracks. LF-NMR showed that CCS/SA (1/3) contained more immobile water content and less free water content, making it more suitable for use as a drug delivery system in tissue engineering [36].

In summary, the thermal stability of the BCS/SA hydrogels was superior to that of the CCS/SA hydrogels. In contrast, the texture, microstructure, and water distribution of the CCS/SA hydrogels were more attractive than those of the BCS/SA hydrogels. The different properties of CCS and BCS and the application of CS/SA hybrid hydrogels as biomaterials in the medical field need to be further investigated.

## 4. Materials and Methods

### 4.1. Materials

Chicken chondroitin sulfate (CCS, CAS 9082-07-9, purity > 90%) was purchased from Qingdao Wantuming Biological Products Co., Ltd., Qingdao, China. Bovine chondroitin sulfate (BCS, CAS 9007-28-7, purity > 90%) was obtained from Qufu Shengjiade Biotechnology Co., Ltd., Qufu, China. Sodium alginate (SA) and anhydrous calcium chloride (analytical purity) were acquired from the Sinopharmaceutical Group Chemical Reagent Co., Ltd., Shanghai, China.

### 4.2. Characterization of CCS and BCS

#### 4.2.1. Particle Size

CCS and BCS aqueous solutions (1 mg/mL) were prepared, and the size distribution of the CCS and BCS particles was determined using a laser scattering particle size analyzer (Nano-ZS90, Malvern Instruments Co., Ltd., Malvern, UK) based on dynamic light scattering with a wavelength of 658 nm, an angle of 90° and a measurement temperature of 25 °C [14].

#### 4.2.2. Transmission Electron Microscopy (TEM)

CCS and BCS aqueous solutions (1 mg/mL) were dripped onto a 200 mesh copper grid coated with a carbon film. The filter paper was dried, and then the samples were observed using a transmission electron microscope (HT7700, Hitachi, Tokyo, Japan) instrument at a magnification of 40,000 under a voltage of 80 kV.

#### 4.2.3. Rheological Measurement

The viscosity of the hydrogel samples was evaluated using a stress-controlled rheometer (MCR302, Anton Paar, Graz, Austria) operated in the frequency scanning mode. CCS and BCS powders were dissolved in distilled water to prepare aqueous solutions with a concentration of 1 mg/mL. Each sample was loaded onto the rheometer plate (40 mm diameter, 1.0 mm gap), the temperature was set to a constant value of 25 °C, the shear rate was increased from 1 to 100 s^−1^, and the sample viscosity shear rate plots were obtained.

#### 4.2.4. Thermogravimetric Analysis (TGA)

Hydrogel samples were freeze-dried in vacuum using an Alpha 1-2 LDplus, Marin Christ, Germany freeze dryer. The freeze-dried samples were analyzed using a TGA, (Mettler Toledo International Co., Ltd., Shanghai, China) instrument. CCS and BCS samples (6 mg) were placed in a crucible, and their temperature was increased from 30 to 500 °C at a rate of 10 °C/min under a nitrogen atmosphere. The changes in mass with temperature were recorded and the thermogravimetric curves of the samples were obtained [38].

#### 4.2.5. Fourier Transform Infrared Spectroscopy (FTIR)

FTIR spectroscopy was used to identify the functional groups of CCS and BCS. An FTIR spectrometer (Nicolet S10, Thermo Fisher Scientific, Waltham, MA, USA) was used to obtain spectra of CCS and BCS in the wavenumber range of 500–4000 cm^−1^ a spectral resolution of 4 cm^−1^ and at a scan rate of 32 scans per minute. The images and data were analyzed using the built-in OMNIC 8.0 software (Thermo Nicolet, Massachusetts, USA) [14].

### 4.3. Preparation of Hybrid Hydrogels

CCS and BCS powders were dissolved in distilled water to prepared aqueous solutions with a concentration of 10%. In addition SA was dissolved in distilled water to prepare an aqueous solution with a concentration of 3%. The CCS and BCS aqueous solutions were mixed with the SA aqueous solution at ratios of 1/3 (*w*/*w*) and 2/3 (*w*/*w*) and the mixtures were stirred for 12 h at (400 rpm, 25 °C) using a magnetic mixer (78-1, Changzhou Danrui Experimental Instrument and Equipment Co., Ltd., Changzhou, China) until they were homogeneous. SA aqueous solution and mixtures of four kinds of CS and SA were poured into molds with a length of 3 cm and a thickness of 3 mm, followed by spraying with a 5% anhydrous calcium chloride aqueous solution to form hydrogels. Thereafter, the hydrogels were washed three times with distilled water to remove residues on their surfaces. The hydrogel samples are denoted as SA, CCS/SA (1/3), CCS/SA (2/3), BCS/SA (1/3), and BCS/SA (2/3).

### 4.4. Characterization of Hybrid Hydrogels

#### 4.4.1. Evaporation Water Loss Rate

Samples of SA, CCS/SA (1/3), CCS/SA (2/3), BCS/SA (1/3), and BCS/SA (2/3) hydrogels (diameter of 3 cm and thickness of 3 mm), were placed in Petri dishes and stored in an incubator at 37 °C. The mass of water lost by hydrogels was recorded at 1, 4, 8, 12, and 24 h, and the evaporation water loss rate was calculated as follows [20]:

$$\mathrm{Evaporation}\;\mathrm{water}\;\mathrm{loss}\;\mathrm{rate}\;(\%)=\mathrm{Evaporation}\;\mathrm{water}\;\mathrm{loss}\;\mathrm{rate}\;(\%)=\frac{m_{1}-{\;m}_{2}}{m_{1}}\times 100,$$

where m_1_ (g) is the initial hydrogel mass and m_2_ (g) is the hydrogel mass loss at different time intervals.

#### 4.4.2. Swelling Rate

Samples of SA, CCS/SA (1/3), CCS/SA (2/3), BCS/SA (1/3), and BCS/SA (2/3) hydrogels (diameter of 3 cm and thickness of 3 mm) were freeze-dried using a vacuum freeze-dryer (Alpha 1-2 LDplus, Marin Christ, Osterode am Harz, Germany). The dry mass of each freeze-dried sample was measured, and then the freeze-dried hydrogel samples were immersed in PBS (50 mM, 10 mL; pH 7.4) and cultured in an incubator at 37 °C. The mass of each sample was recorded at 1, 4, 8, 12, and 24 h, and each measurement was performed afterwards by removing excess water from the sample surface [39]. The swelling rate was calculated as follows:

$$\mathrm{Swelling}\;\mathrm{rate}\;(\%)=\frac{m_{1}-{\;m}_{2}}{m_{2}}\;\times \;100,$$

where m_1_ (g) is the mass of hydrogel soaked in PBS for different times, and m_2_ (g) is the mass of lyophilized hydrogel.

#### 4.4.3. Thermogravimetric Analysis (TGA)

Samples of SA, CCS/SA (1/3), CCS/SA (2/3), BCS/SA (1/3), and BCS/SA (2/3) hydrogels were freeze-dried using a vacuum freeze-dryer (Alpha 1-2 LD Plus, Marin Christ, Germany). Each lyophilized sample (6 mg) was placed in an alumina crucible, and an empty alumina crucible was used as the blank. The temperature of the samples was increased from 50 to 500 °C at the rate of 10 min/°C under a nitrogen atmosphere, and a dynamic test was performed. The thermogravimetric curves of the samples were recorded continuously [38].

#### 4.4.4. Fourier Transform Infrared Spectroscopy (FTIR)

The FTIR spectra of the SA, CCS/SA (1/3), CCS/SA (2/3), BCS/SA (1/3), and BCS/SA (2/3) hydrogels were obtained using an FTIR spectrometer (Nicolet S10, Thermo Fisher Scientific, USA) in the wavenumber range of 4000–500 cm^−1^ at a spectral resolution of 4 cm^−1^ and a scan rate of 32 times per minute. The FTIR data were analyzed using the OMNIC 8.0 software [14].

#### 4.4.5. Texture Analysis

The texture of the SA, CCS/SA (1/3), CCS/SA (2/3), BCS/SA (1/3), and BCS/SA (2/3) hydrogels was evaluated using a texture analyzer (TA.XT PlusC, Stable Micro Systems, Godalming, UK) at room temperature. The probe and experimental parameters were as follows: probe model *P*/0.5R, pre-test speed 2.0 mm/s, test speed 1.0 mm/s, post-test speed 2.0 mm/s, pressing distance 1.0 mm, and triggering force 5.0 g [16].

#### 4.4.6. Scanning Electron Microscopy (SEM)

Samples of the SA, CCS/SA (1/3), CCS/SA (2/3), BCS/SA (1/3), and BCS/SA (2/3) hydrogels were added to a glutaraldehyde solution (volume fraction of hydrogel of 2.5%), allowed to rest overnight at 4 °C, and then rinsed with a 0.1 mol/L phosphate buffer solution (pH 7.4) six times for 10 min each. Thereafter, the hydrogel samples were dehydrated for 15 min with ethanol at volume fractions of 50, 60, 70, 80, 90 and 100%. Subsequently, the samples were rinsed with tert-butyl alcohol three times, for 30 min each. The dehydrated hydrogel samples were lyophilized, and gold was sprayed on their surfaces using a vacuum ion sputtering coater. Lastly, the surfaces of the hydrogel samples were observed using an SEM (7500F, JEOL Electronics, Tokyo, Japan) instrument at a magnification of 1500× under a voltage of 2 kV [40].

#### 4.4.7. Low-Field Nuclear Magnetic Resonance (LF-NMR)

The transverse relaxation times (T_2_) of the SA, CCS/SA (1/3), CCS/SA (2/3), BCS/SA (1/3), and BCS/SA (2/3) hydrogels were measured using an LF-NMR (MicroMR20-025, Suzhou Niumag Analytical Instrument Co., Ltd., Shanghai, China) instrument, and the SIRT algorithm was used for 1,000,000 iterative fittings. The SF, O1, P1, P2, TD, TW, NS, and NECH experimental parameters were 20 MHz; 829.356 kHz; 8 μs; 16 μs; 720016; 5000 ms; 4; and 12,000, respectively.

### 4.5. Statistical Analysis

Analysis of variance and Tukey’s test were conducted using the SPSS 19.0 software (IBM, Armonk, NY, USA), and the results were used to determine the statistical significance (*p* < 0.05) of the data. Data are presented as means and standard deviations. All measurements were repeated three times.

## Data Availability

Not applicable.
